# Second Operation for Extra-Uterine Pregnancy

**Published:** 1913-04

**Authors:** 


Second Operation for Extra-Uterine Pregnancy.—Stuart McGuire of Richmond, *Old Dominion Journal,* January, 1913, in a series of 35 cases, had five requiring a second operation, six that bore normal children, the remainder not being traceable. Richard R. Smith of Grand Rapids tabulated 2,998 cases of operation for ectopic gestation with recurrence in 113—3/8%. Three of Smith's 30 personal cases subsequently bore children normally. In discussing the difficult problem of radical operation, sterilizing the patient, McGuire sagely remarks that all of the women who had a repetition of the ectopic pregnancy favored it and all who had borne normal children disapproved, and he emphasizes that the patient before operation is not in a position to judge correctly. The operator must therefore decide according to social conditions, as age, desire for offspring, number of previous children, and also with regard to the pathologic states encountered.

